# Tickborne Relapsing Fever Outbreak at an Outdoor Education Camp — Arizona, 2014

**Published:** 2015-06-19

**Authors:** Jefferson M. Jones, Mare Schumacher, Marie Peoples, Nina Souders, Kimberly Horn, Lisa Fox, Michele Scott, Shane Brady, Joli Weiss, Ken Komatsu, Nathan Nieto

**Affiliations:** 1 Epidemic Intelligence Service, CDC, Atlanta, Georgia; 2 Maricopa County Department of Public Health, Phoenix, Arizona; 3 Arizona Department of Health Services; 4 Coconino County Public Health Services District, Flagstaff, Arizona; 5 Flagstaff Medical Center, Flagstaff, Arizona; 6 Phoenix Children’s Hospital, Phoenix, Arizona; 7 Northern Arizona University, Flagstaff, Arizona

Tickborne relapsing fever (TBRF) is a bacterial infection characterized by recurring episodes of fever, headache, muscle and joint aches, and nausea. In North America, TBRF primarily is caused by *Borrelia hermsii* spirochetes transmitted by *Ornithodoros hermsii* ticks (1). Once infected, these soft ticks are infectious for life (2) and transmit the spirochete to sleeping humans quickly (possibly within 30 seconds) during short feeds (15–90 minutes) (1–3). On August 10, 2014, the Coconino County Public Health Services District in Arizona was notified by a local hospital that five high school students who attended the same outdoor education camp had been hospitalized with fever, headache, and myalgias. Hantavirus infection initially was suspected because of reported exposure to rodent droppings, but after detecting spirochetes on peripheral blood smears from all five hospitalized students, TBRF was diagnosed. The camp was instructed to close immediately, and the health department, in collaboration with local university experts, investigated to identify additional cases, determine the cause, and prevent further infections. A total of 11 cases (six confirmed and five probable) were identified.

Camp staff members and attendees were interviewed during August 11–14. Medical records of the five hospitalized patients were reviewed, and the campsite was inspected for evidence of rodent or tick infestation. Consistent with the Arizona Department of Health Services case definition, a probable case was defined as an illness with at least three of the four major TBRF signs and symptoms (fever, chills, myalgias, and headache) without laboratory testing in a person attending the camp during August 1–3, 2014. A case was confirmed by visualization of spirochetes in an attendee’s blood smear or by *Borrelia hermsii* isolation by culture.

During August 1–3, a total of 45 persons (39 high school football players and six adult coaches) attended a school-run outdoor education camp located in a wooded area in Coconino County. Thirty-one (69%) of the 45 persons at the camp were interviewed. Six confirmed cases (four by visualization of spirochetes on blood smear and isolation and two by visualization of spirochetes alone) and five probable cases were identified (attack rate: 24%). Ten patients were students aged 15–17 years, and one was a coach aged 33 years.

All six persons with confirmed TBRF and four of the five persons with probable TBRF had slept in the camp’s main cabin. Using the earliest date when common exposure might have occurred (August 1), the median incubation period was 6 days (range = 2–10 days). All six of the persons with confirmed TBRF had fever, headache, myalgias, and arthralgias; all five of those with probable TBRF had fever, headache, and myalgias (Table). Among the six with confirmed TBRF and known laboratory values, five had thrombocytopenia (platelets <150/*μ*L); four had decreased albumin; and three had elevated transaminases. Eight of the 11 patients were treated with doxycycline and had no known major complications; the six patients with confirmed TBRF were treated with 100 mg doxycycline twice daily for 7–10 days. Attempts to obtain clinical and laboratory information on patients with probable TBRF were unsuccessful.

The investigation revealed that, during July 17–24, professional pest controllers had performed rodent-proofing activities at the main cabin; however, no acaricides (pesticides that kill ticks and mites) were applied. On August 12 and 28, the public health team inspected the cabin and found evidence of rodents and soft tick infestation, including rodent nesting material in a woodpile in a crawl space beneath the cabin, squirrel droppings in a chimney crevasse, and one live and one desiccated *Ornithodoros hermsii* tick. Among four chipmunks (*Tamias dosalis*) trapped on August 12, two were documented with *B. hermsii* by positive quantitative polymerase chain reaction. Testing of one soft tick for *B. hermsii* was negative. Camp management was provided written instructions regarding rodent-proofing and acaricide application. The camp reopened after recommendations were implemented; no additional cases have been identified.

During 1982–2013, a total of 22 TBRF cases (0–3 cases annually) were reported in Arizona residents. This 2014 outbreak of TBRF with 11 confirmed and probable cases is the largest recorded in Arizona since 1990. Health care providers and public health professionals should be aware that TBRF is a possible cause of febrile illness among patients with a travel history to areas where TBRF is endemic, particularly if they have slept in a rustic cabin (1). These findings suggest that pest control companies and cabin owners might benefit from education regarding prevention of tickborne diseases, including sleeping off the floor and away from walls, applying insect repellent on skin and clothing, and rodent-proofing.

To eliminate tick populations in a building, it is important to consider acaricide spraying concurrently with rodent-proofing because removing rodents from buildings can result in ticks losing their primary source of food and feeding on humans as an alternative (2).

## Figures and Tables

**TABLE:** Number of signs and symptoms of patients with confirmed or probable tickborne relapsing fever in an outbreak associated with an outdoor education camp — Arizona, 2014

Sign or symptom	Total no. (N = 11)	Confirmed no. (n = 6)	Probable no. (n = 5)
Fever	**11**	6	5
Headache	**11**	6	5
Myalgias	**11**	6	5
Arthralgias	**10**	6	4
Abdominal pain	**7**	5	2
Fatigue	**6**	2	4
Vomiting	**5**	4	1
Cough	**4**	2	2
Dizziness	**3**	3	0
Syncope	**2**	2	0
Rash	**2**	2	0
